# Intimate partner violence during pregnancy and risk of still birth in hospitals of Tigray region Ethiopia

**DOI:** 10.1186/s13052-020-00857-w

**Published:** 2020-07-27

**Authors:** Kahsay Zenebe Gebreslasie, Solomon Weldemariam, Gelawdiyos Gebre, Mihret-Ab Mehari

**Affiliations:** Mekelle University, College of Health Science, Mek’ele, Ethiopia

**Keywords:** Intimate partner, Violence, Pregnancy, Still birth

## Abstract

**Background:**

Pregnancy may represent a time of exceptional vulnerability to intimate partner violence because of changes in women’s conditions. Despite the fact that intimate partner violence during pregnancy confers considerable risk to the health of the woman and her fetus, data regarding to association of stillbirth and intimate partner violence is lacking in Tigray region. The objective of this study is to assess intimate partner violence during pregnancy and its association with still birth among postpartum mothers in hospitals in Tigray Region of Ethiopia.

**Methods:**

Cross-sectional study design was used to assess 648 women about intimate partner violence during pregnancy and its association with still birth. Simple random sampling technique was employed to select health facilities and systematic sampling was used to select the study participants. Data was entered by using Epi info version 3.5.1 and analyzed using SPSS version 20. Logistic regression analysis was done to assess the association between exposure to intimate partner violence during pregnancy and stillbirth while adjusting for possible confounders.

**Results:**

The prevalence of still birth was 3.6%in this study population. There was a statistically significant association between exposure to intimate partner violence during pregnancy and still birth. Pregnant women who were exposed to intimate partner violence during pregnancy were three times more likely to have still birth 3.3(95% CI: 1.1–9.7) as compared to those who were not exposed. Another important factor associated with stillbirth was low birthweight 16.7(95% CI,6–46).

**Conclusions:**

The prevalence of still birth in this study was high. Women who subjected to intimate partner violence during pregnancy had greater risk of having stillbirth baby.

## Background

Intimate partner Violence (IPV) is defined as a psychological, sexual and physical harm by a current or former partner [1]. Intimate partner violence is a public health problem at national and global level [2, 3]. IPV has wide reaching negative impact on a woman’s autonomy, self-esteem, ability care for her family and her ability participate in social activities [4, 5].

A multi-country study by the World Health Organization shows that 15 to 71% of women experience physical and/or sexual in their lifetime [6]. Domestic violence is a common and tolerated practice in both urban and rural part of Ethiopia. An Ethiopian study elucidated that nearly three out of four (71%) women are subjected to IPV in their lifetime [7].

The prevalence of IPV among pregnant mothers ranged from 13.5% in Uganda to 2% in Australia [8]. Study from multi country also shown the prevalence of IPV during pregnancy was range from 1 to 28% [5]. In addition to this, a systemic review of research conducted in various African countries indicated that the overall prevalence of intimate partner violence during pregnancy ranges from 2.3 to 57.1% [9].

In Ethiopia, IPV during pregnancy was found to range from 4% (merely physical violence) to 8% (all forms of IPV) [7, 10]. Study from Northwest Ethiopia revealed that the occurrence of IPV during pregnancy was 25.4% [5].

According to Ethiopia’s demographic health Survey data in 2016, perinatal mortality rate was 33 per 1000 pregnancies [10].

According to 2014 new born action plan global multi partner movement to end preventable still birth, the target was set to reduce the magnitude of stillbirth to less than 12 per 1000 births[(11]. A study undertaken in 157 countries revealed that the estimated stillbirth rate was 18.4/1000 births. Of the 2.6 million stillborn babies delivered in 2015, 98% occurred in low and middle income countries [11].

The risk of IPV is high among women of reproductive age and this is mainly attributed to changes in physical, social, emotional or financial status during pregnancy. Pregnancy puts a woman at greater vulnerability to intimate partner violence [12]. Consequently, IPV during pregnancy worsens maternal condition and predisposes to adverse fetal and neonatal outcomes [13, 14]. Some existing research indicates that there may be a statistically significant association of IPV during pregnancy and stillbirth [3, 15]. This might be due to the impact of IPV on a women’s life style, physical and psychological health [16]. Some studies however do not show an association between IPV violence and stillbirth [7]. This may be less about lack of association and more about grave underreporting of IPV in Africa.

In Ethiopia, particularly in study area, there are no studies looking at the association between IPV during pregnancy and stillbirth. Therefore, this study aims to bridge this gap.

## Methods

### Study setting

The Tigray region is located 783 km away from Addis Ababa, capital city of Ethiopia. According to 2007 census, the population of Tigray region was estimated to be 4,316,988. Women of child bearing age (15–49) comprise 251,650 of the population. According to 2015 Tigray regional health bureau annual report, there are a total of one specialized referral hospital, 15 general hospitals, 22 primary hospitals, 204 health centers and 712 health posts and three private hospitals. There are 51 doctors with specialty training in obstetrics and gynecology, 87 general practitioners, 3092 nurses and 792 midwives in the region. The study was conducted from November 2017–June 2018. Institutional based cross-sectional study was used.

### Sample

The study population was all women who gave birth in hospitals within the Tigray region. Women who are unable to hear were excluded from the study.

There are 41 hospitals (1 specialized hospital, 15 general hospitals, 22 primary hospitals and 3 private hospitals) which provide delivery service in the study area. Health facilities were stratified in to private and public hospitals. For the purposes of this study, one private hospital and eight public hospitals were selected by simple random sampling technique. Participants from each selected health facilities were sampled by systematically. Every 3rd postpartum women were included until the required sample size was reached. Consecutive participant was included if the selected participant was not eligible. Average client load for each hospital was assessed using the patient flow three months’ prior to data collection period and proportional allocation to each hospital was made based on their respective quarterly client flow.

To calculate the sample size, we used available data that indicates 25.8% prevalence of IPV during pregnancy in Ethiopia [14] and 95% confidence interval, 5%margin of error, design effect 2 and expected non response rate 10%. Based on this, the calculated sample size was 648.

Data on socio-demographic characteristics of participants (age, residence, religion, educational status, marital status and occupational status) and obstetrics characteristic (mode of delivery, PROM, hypertension, APH, ANC, follow up, apgar score, preterm birth, low birth weight and whether the was desired pregnancy/not) were collected through interviews and chart view.

### Instrument

Data was collected using a structured questionnaire that was administered to post-partum women by trained staff. Staff involved in administering the questionnaires included nine midwives (diploma level training) who were supervised four Midwives (bachelor level training). Training was given to both data collectors and supervisors about the aim of the study, procedures, how to approach the study participants and data collection techniques.

#### Intimate partner violence

Maternal exposure to IPV was determined through the question: “when you were pregnant with this child, did your current partner or boyfriend do any of the following things to you? The lists of potential offences were as follows: Physical violence: slapped, pushed or shoved, hit with fist or something else that could hurt her, trauma to the abdomen, choked or burnt on purpose, used or threatened to use knife, gun or weapon. Emotional violence: insult, humiliation, intimidation on purpose, verbal threats. Sexual violence: Forced into sexual intercourse when you did not want, had sexual intercourse when you did not want to because you were afraid of what partner might do, forced to do something sexual that you found degrading or humiliating. **Stillbirth:** is typically defined as fetal death at or after 28 weeks of pregnancy. It results in a baby born without signs of life.

### Procedure

Questionnaire was prepared first in English and then translated into Tigrigna and back translated to English by language expert to ensure the accuracy of the information.

Data on still birth was collected from the medical charts and direct interviews via questionnaires. Outcomes of interest for this analysis pertained directly to neonatal outcomes and were obtained through chart review within 72 h of delivery. Birth weight (g) and gestational age (weeks) were taken directly from the chart. Low birth weight was assigned if the neonate weighed < 2500 g, and preterm birth was considered if the neonate was born at < 37 completed weeks of gestation and > 28 weeks. Gestational age was computed (dated) from either first trimester ultrasound or reliable last menstrual period.

An ethical approval for the study was obtained from Mekelle University College of health science health research ethics review committee. Permission letter was obtained from regional health office and was presented to selected hospitals. Written consent was taken from each participant before the starting of data collection and for those women who are under age, written consent was obtained from their parent. Since IPV is sensitive issue the interviews were conducted in a private room. Confidentiality was maintained throughout the study. In addition, participants were told that they have the right not to participate and/or could withdraw from the study at any point.

### Data process and analysis

Double data entry was done by using Epi info version 3.5.1 and exported to SPSS version 20 software package for analysis. Experience of any physical, sexual or emotional violence was considered if a woman reported being exposed to at least one of the acts of violence exerted by her partner while she was a pregnant for current neonate.

To estimate the association between maternal exposure to intimate partner violence and risk of still birth, logistic regression analyses were performed and odds ratios (OR) with 95% confidence intervals (CI) were calculated. Multivariable logistic regression analysis was performed where intimate partner violence plus other covariates that could influence still birth such as age, educational level, occupation during pregnancy and alcohol intake etc. The degree of association between independent and dependent variables were assessed using odds ratio with 95% confidence interval.

## Results

### Socio-demographic characteristics

A total of 647 participants took part in this study with a response rate of 99.8%.Out of the total respondents, 458 (70.78%) of them were urban residents. The mean age of the respondents was 27 ± 6 years. Majority of respondents 530(81.9%), were between ages 20–35 years old although a few were younger. Most participants were married (*N* = 610; 94.28%) were married. Out of the participants, nearly half were housewives (*N* = 301; 46.5%) (Table 1).

**Table 1:** Socio-Demographic characteristics of respondents, Tigray, North, Ethiopia, 2018

Variable		Frequency	Percent
**Residence**	Urban	458	70.78
	Rural	189	29.22
**Age**	≤19	50	7.7
	20–34	530	82
	≥35	67	10.3
**Religion**	Orthodox	581	89.8
	Muslim	66	10.2
**Educational Status**	Unable to read &write	108	16.7
	Read and write	44	6.8
	Primary education	175	27
	Secondary education and college	211	32.6
	Diploma and above	109	16.8
**Marital Status**	Married	610	94.3
	Single	37	5.7
**Occupational status**	Housewife	301	46.5
	Merchant	71	11
	Farmer	127	19.6
	Private employee	42	6.5
	Governmental employee	95	14.7
	Others	11	1.7

### Obstetrics characteristics of the participants

A quarter of the women (*N* = 155; 24%) delivered via cesarean section. Similarly, a quarter of the women (25%) experienced premature rupture of membrane, 66 (10.2%) experienced hypertension and 35 (5.4%) had antepartum hemorrhage (APH). Among these participants, 611(94.4%) had at least one antenatal care (ANC) follow up during their last pregnancy. In this study population, 42 (6.5%) of the women admitted that their index pregnancy was unwanted. Data on pregnancy outcome shows that 70 (10.8%) women delivered before reaching term and 120 (18.5) babies were low birth weight. The magnitude of still birth in this study was 23 (3.6%) (Table 2).

**Table 2:** Obstetrics characteristics of respondents, Tigray, North, Ethiopia, 2018

Variable		Frequency	Percentage
Mode of delivery	Vaginal	492	76
	C/S	155	24
PROM	Yes	162	25
	No	485	75
Hypertension	Yes	66	10.2
	No	581	89.8
APH	Yes	35	5.4
	No	612	94.6
ANC follow up	Yes	611	94.4
	No	36	5.6
Still birth	Yes	23	3.6
	No	624	96.4
Pregnancy wanted	Yes	605	93.5
	No	42	6.5
Preterm delivery	Yes	70	10.8
	No	577	89.2
Low birth weight	Yes	120	18.5
	No	527	81.5

### Substance use

Of the total participants, 288 (44.5%) women admitted that they ingested alcohol during pregnancy sometimes and 10(1.6%) ingested chat (a stimulant leaf) while they were pregnant with the index neonate. Three (0.5%) women smoked while pregnant.

### Types of intimate partner violence

Around 47 (7.3%) women experienced intimate partner violence during their index pregnancy in which 22 of them were subjected to physical violence, 39 of them experienced sexual violence and the remaining 8 women were subjected to psychological violence.

### Factors associated with still birth

In our study, women who were subjected to intimate partner violence during pregnancy are 3.3 times (AOR = 3.3; 95% CI: 1.1–9.7) more likely to have stillborn baby than who did not experience IPV during pregnancy.

Low birth weight was also significantly associated with still birth. Babies with low birth weight have 16.7 times (AOR = 16.7; 95% CI:6–46) risk of still birth as compared with babies’ weight greater than or equal to 2.5 kg. Having unwanted pregnancy and preterm birth were significant associated with still birth in bivariate analysis but is has no association in multivariate analysis (Table 3).

**Table 3:** Bivariate and multivariate logistic regression analyses of still birth by socio demographic variables, obstetrics related variables and intimate partner violence during pregnancy

Variables		Still birth		COR(95% CI)	AOR(95% CI)
		Yes	No		
Marital status	Marriage	20	590	.38 (0.109–1.35)	
	Single	3	34	1:00	
Resident	Urban	13	445	.52 (0.22–1.2)	
	Rural	10	179	1:00	
Religion	Orthodox	21	560	1.2 (0.27–5.2)	
	Muslim	2	64	1:00	
Age	> 19	4	46	1.37(.32–5.7)	
	20–35	15	515	.45 (0.14–1.4)	
	> 35	4	63	1:00	
IPV	Yes	6	41	5 (1.87–13.4)	3.3 (1.1–9.7)
	No	17	583	1:00	1:00
Hypertension	Yes	2	64	.83 (0.19–3.6)	
	No	21	560	1:00	
APH	Yes	3	32	2.77 (0.78–9.8)	
	No	20	592	1:00	
PROM	Yes	4	158	.62 (0.2–1.8)	
	No	19	466	1:00	
Habit of alcohol intake	Never	13	346	1.04 (0.45–2.4)	
	Sometimes	10	278	1:00	
Pregnancy wanted	Yes	19	586	1:00	
	No	4	38	3.24 (1.05–10)	2.3 (0.72–7.7)
ANC follow up	Yes	22	589	1.3 (0.17–9.9)	
	No	1	35	1:00	
Birth weight	> = 2.5 kg	5	522	1:00	1:00
	< 2.5 kg	18	102	18.4 (6.6–50.7)	16.7 (6–46)
Gestational age	> = 37 week	13	564	1:00	
	< 37 week	10	60	7.23 (3–17)	

## Discussion

This study which assessed the association between IPV during pregnancy and stillbirth provided new and important information that has been missing from research in low income countries like Ethiopia. The magnitude of intimate partner violence during pregnancy is concerning and can have important implications on neonatal outcome. This study revealed that the magnitude of intimate partner violence during their index pregnancy was 7.3%. This is lower than study done in Tanzania (30%), Vietnam (32.5%) and Ethiopia hosanna (23%) [7, 14, 17]. These disparities in the reported prevalence rates might be attributed to study area and time differences. Most of the studies with higher prevalence were done many years ago and it is plausible that there has been some improvement in awareness about the dangers of IPV during pregnancy. However, it is also possible that there may be under reporting of IPV in our study population.

In this study the prevalence of still birth was found to be 3.6%. Our finding is similar with the findings from Tanzania [18] and Zimbabwe [19] where 3.5 and 5.6% of women had still birth respectively. But this study finding is significantly higher than that reported from central Vietnam (0.97%) and the recommended goal of 1.2% by the 2014 newborn action plan.

[9, 20]. This difference might be accounted by study area difference or difference in accessibility to prenatal or emergency obstetric care services.

This study found that IPV has significant association with still birth. This finding is in line with a research done in Columbia, South Carolina and California which indicated that women who experienced IPV during pregnancy have increased risk of still birth [21, 22]. IPV can lead to still birth either by direct (trauma) or indirect impact (such as decreased nutritional intake due to psychosocial trauma etc). In addition to this, women who experience sexual violence may be exposed to HIV and sexually transmitted diseases that can impact neonatal outcome. Finally, IPV can affect women’s ability to access antenatal care.

In this study still birth has association with low birth weight. Babies delivered with low birth weight increased the risk of still birth by sixteen times. This finding is supported by research done in north Tanzania and peri-urban District in Ghana; being low birth weight increase the risk of still birth by more than nine times [18, 23]. Fetus with low weight may have a high risk of death due to their immature respiratory system [18].

### Limitation of the study

Since IPV during pregnancy is sensitive and self-reported, there is a risk of under reporting. Given that this is a cross-sectional rather than a prospective study, it is not possible to establish the temporal relation between IPV and stillbirth.

## Conclusion

This study shows that still birth is high in this population and intimate partner violence during pregnancy has significant association with pregnancy outcome, namely still birth. It is important that healthcare providers involved in maternal care as well as the federal ministry of health prioritize formulating a protocol for screening intimate partner violence during pregnancy to reduce still birth.

## Data Availability

The data supporting the findings of this article can be provided if requested us.

## Abbreviations

EDHS: Ethiopia Demographic Health Survey
IPV: Intimate Partner Violence
SGA: Small for Gestation Age
PROM: Premature Rapture of Membrane
ANC: Antenatal Care
APH: Antepartum Hemorrhage
